# Association between admission heart rate and in-hospital mortality in patients with acute exacerbation of chronic obstructive pulmonary disease and respiratory failure: a retrospective cohort study

**DOI:** 10.1186/s12890-024-02934-w

**Published:** 2024-03-05

**Authors:** Ruoqing Zhou, Dianzhu Pan

**Affiliations:** Department of Respiratory Medicine, The First Affiliated Hospital of Jinzhou Medical University, Jinzhou, China

**Keywords:** Admission heart rate, Acute exacerbation of chronic obstructive pulmonary disease, In-hospital mortality, Respiratory failure

## Abstract

**Background:**

Acute exacerbation of chronic obstructive pulmonary disease (AECOPD) combined with respiratory failure (RF) is a chronic respiratory disease that seriously endangers human health. This study aimed to specifically evaluate the relationship between admission heart rate (AHR) and in-hospital mortality in patients with combined AECOPD and RF to better inform clinical treatment.

**Methods:**

This retrospective cohort study included 397 patients admitted to a Chinese hospital between January 2021 and March 2023. The primary outcome measure was all-cause in-hospital mortality. Multivariate logistic regression analyses were performed to calculate adjusted hazard ratios (OR) with corresponding 95% confidence intervals (CI), and curve fitting and threshold effect were performed to address nonlinear relationships.

**Results:**

In total, 397 patients with AECOPD/RF were screened. The mean (± SD) age of the study cohort was 72.6 ± 9.5 years, approximately 49.4% was female, and the overall in-hospital mortality rate was 5%. Multivariate logistic regression analysis and smooth curve fitting revealed a nonlinear association between AHR and in-hospital mortality in the study population, with 100 beats/min representing the inflection point. Left of the inflection point, the effect size (OR) was 0.474 (95% CI 0.016 ~ 13.683; *p* = 0.6635). On the right side, each 1 beat/min increase in AHR resulted in an effect size (OR) of 1.094 (95% CI 1.01 ~ 1.186; *p* = 0.0281).

**Conclusions:**

Results of the present study demonstrated a nonlinear relationship between AHR and in-hospital mortality in patients with AECOPD/RF. When AHR was < 100 beats/min, it was not statistically significant; however, AHR > 100 beats/min was a predictor of potential mortality, which increased by 9.4% for every 1 beat/min increase in AHR.

## Background

Chronic obstructive pulmonary disease (COPD) is currently one of the leading causes of death worldwide [1, 2] Approximately 3 million individuals died of COPD in 2012, accounting for 6% of all deaths globally, and COPD is the main cause of chronic mortality and morbidity worldwide [3].Acute exacerbation of COPD (AECOPD) is defined as dyspnea, cough, and sputum aggravation within 14 days, and may be accompanied by tachypnea and/or tachycardia [4]. Based on healthcare resource use, AECOPD may be classified as mild, moderate, or severe. Patients with mild disease can self-treat by increasing the use of currently prescribed medications, and those with moderate disease require systemic steroids or antibiotics. However, individuals with severe disease often experience rapid deterioration and require hospitalization [5]. AECOPD is one of the most common causes of hospital admission, and those with frequent aggravation experience reduced quality of life and an accelerated decline in lung function [6]. Respiratory failure (RF) is a complication that contributes the most to the high mortality and poor prognosis of patients with AECOPD [7].

Admission heart rate (AHR) was defined as the first available heart rate measured from the initial admission [8]. Some studies have documented that elevated AHR is an independent predictor of both short- and long-term mortality after discharge from hospital for acute myocardial infarction [9–14]. Patients with COPD die more frequently from cardiovascular diseases than from respiratory disease [15–17]. Faster heart rates are associated with health risks, and individuals with COPD have a higher cardiovascular risk [18]. Further studies are warranted to confirm these findings, and a detailed analysis of the relationship between AHR and in-hospital mortality in patients with AECOPD and RF is warranted.

## Methods

The present investigation was a single-center retrospective analysis including patients > 40 years of age, who were diagnosed with AECOPD and RF at the First Affiliated Hospital of Jinzhou Medical University (Liaoning Province, China) between January 2021 and March 2023. Information used in the study included patient demographics, vital signs, laboratory investigations, diagnostic and treatment information, and outcomes. This study was performed in accordance with the principles of the Declaration of Helsinki and was approved by the Ethics Review Board of the First Affiliated Jinzhou Medical University.

All patients admitted to hospital with a primary diagnosis of AECOPD according to the Global Initiative for Chronic Obstructive Lung Disease (i.e., “GOLD”) criteria were enrolled if they were diagnosed with RF confirmed by arterial blood gas analysis on admission. Patients < 40 years of age, those with a history of multiple admissions, those for whom heart rate data were not available, patients with AHR < 35 beats/min and, finally, individuals with incomplete data, were excluded.

### Data collection

All patient demographic information and laboratory data were extracted from the electronic medical system of the First Affiliated Jinzhou Medical University. Patient demographics including age, sex, smoking status, length of hospital stay, use of inhaled corticosteroids (ICS) and ventilation, and comorbidities, with special emphasis on cardiopulmonary disease, hypertension, diabetes mellitus (DM), coronary heart disease (CHD), cor pulmonale, heart failure, and RF type, identified from medical record review, were recorded. Clinical data, including vital signs (respiratory rate [RR], systolic and diastolic blood pressure),NEWS score(respiratory rate, oxygen saturations, systolic blood pressure, pulse, level of consciousness and temperature) were recorded on admission to the ward. Initial laboratory results obtained within 24 h of the hospital visit were collected, including arterial blood gases (pH, partial pressure of oxygen [*Pa*O_2_], and partial pressure of carbon dioxide [*Pa*CO_2_]), general biochemical tests (blood urea nitrogen [BUN], albumin [ALB], creatinine, potassium, and sodium), and routine hematology tests (white blood cell [WBC] count, percent neutrophils [NEU%], platelets, hemoglobin [Hb]). Comorbidities were diagnosed on the basis of patient medical history or medication use. The primary outcome measure was all-cause, in-hospital mortality, which was defined as death during hospitalization.

### Statistical analysis

All statistical tests were two-tailed and differences with *P* < 0.05 were considered to be statistically significant. All analyses were performed using the R package (http:// www.R-project.org, R Foundation for Statistical Computing, Vienna, Austria) and Free Statistics software version 1.8 [19]. Categorical variables are expressed as number (percentage), while continuous variables are expressed as mean ± standard deviation (SD) or median (interquartile range). The chi-squared test or Fisher’s exact test for categorical variables was used to compare the characteristics of the study participants among the outcome groups, and differences in continuous variables were tested using analysis of variance or the rank-sum test, as appropriate. Multivariate logistic regression analyses were performed to assess the independent association between AHR and in-hospital mortality using 4 models in the regression analysis, as follows: model 1, not adjusted; model 2 was adjusted for age, sex, smoking status, and NEWS score; model 3 was adjusted for the variables in model 2 plus RF type, DM, high blood pressure, cor pulmonale, and CHD; model 4 was adjusted for model 3 plus *Pa*O_2_, *Pa*CO_2_, NEU%, Hb, platelets, ALB, BUN, creatinine, potassium, and sodium. Smooth curve fitting was used to explore the relationship between AHR and in-hospital mortality, Threshold effect analyses were conducted to assess the ability of AHR levels to predict in-hospital mortality. Interaction and stratified analyses were also performed according to age, sex, RF type, high blood pressure, cor pulmonale, and whether ICS were used.

## Results

### Baseline characteristics of the study population

**Fig. 1:**
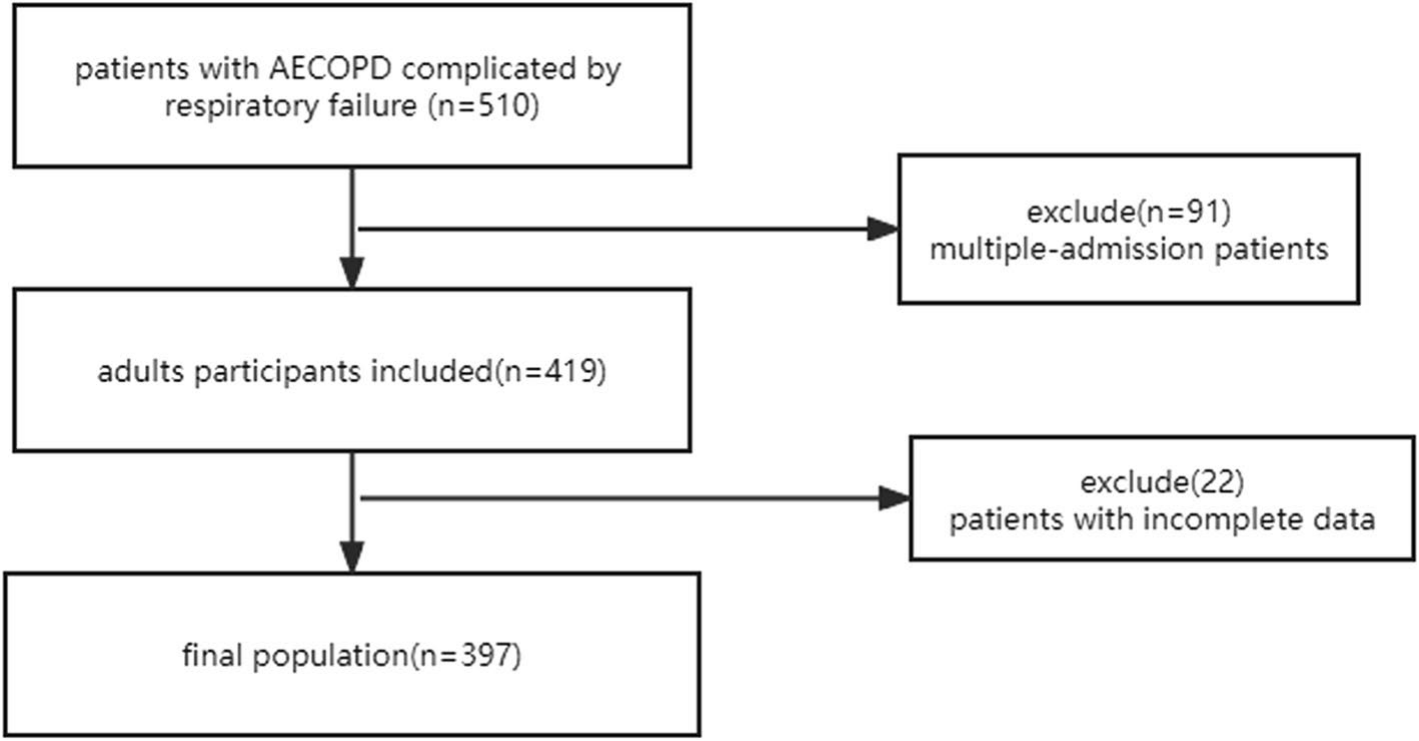
Flowchart of the study cohort

In total, 510 patients with AECOPD and RF were admitted to the First Affiliated Hospital of Jinzhou Medical University between January 2021 and March 2023, of whom 397 were eligible for inclusion in the present study (Fig. 1). Demographic information and characteristics of these patients at the time of admission are summarized in Table 1. The mean age of the study cohort was 72.6 ± 9.5 years, approximately 49.4% was female, and the over all in-hospital mortality rate was 5%. History of disease among the study population included the following: hypertension (*n* = 135); DM (*n* = 43); cor pulmonale (*n* = 187); heart failure (*n* = 167); type I RF (*n* = 131); and type II RF (*n* = 266). ICS were used by 261 patients and 199 used a ventilator. The hospital non-survivor group was older, and exhibited higher WBC count, NEU%, BUN, and creatinine than the hospital survivor group (*p* < 0.05), and shorter length of hospital stay and lower ALB levels (*P* < 0.05). Patients in the hospital non-survivor group exhibited higher AHR (107.0 ± 21.8 versus 96.2 ± 18.2 beats/min; *P *= 0.011) than those in the survivor group.

**Table 1:** Baseline characteristics of participants

Variables	Total (*n *= 397)	survivor (*n* = 377)	non-survivor (*n* = 20)	*p*
Age, years	72.6 ± 9.5	72.3 ± 9.5	77.8 ± 7.3	0.011
Sex, n (%)				0.39
Male	201 (50.6)	189 (50.1)	12 (60)	
Female	196 (49.4)	188 (49.9)	8 (40)	
Los, days	10.0(7.0,12.0)	10.0(7.0,13.0)	4.0 (2.0,8.8)	< 0.001
Smoking status, n (%)				0.648
Never	178 (44.8)	171 (45.4)	7 (35)	
Former	136 (34.3)	128 (34)	8 (40)	
Now	83 (20.9)	78 (20.7)	5 (25)	
SBP, mmHg	136.5 ± 24.5	136.6 ± 24.1	135.6 ± 31.8	0.866
DBP, mmHg	80.9 ± 16.1	81.0 ± 15.7	79.2 ± 21.9	0.632
Respiratory rate,(times/min)	21.8 ± 2.5	21.8 ± 2.6	21.8 ± 1.8	0.944
Heart rate, (bpm)	96.8 ± 18.5	96.2 ± 18.2	107.0 ± 21.8	0.011
ICS, n (%)				0.003
No	136 (34.3)	123 (32.6)	13 (65)	
Yes	261 (65.7)	254 (67.4)	7 (35)	
ventilator, n (%)				0.006
No	198 (49.9)	182 (48.3)	16 (80)	
Yes	199 (50.1)	195 (51.7)	4 (20)	
Respiratory failure type, n (%)				0.494
I	131 (33.0)	123 (32.6)	8 (40)	
II	266 (67.0)	254 (67.4)	12 (60)	
DM,n (%)				1
No	354 (89.2)	336 (89.1)	18 (90)	
Yes	43 (10.8)	41 (10.9)	2 (10)	
HBP, n (%)				0.923
No	262 (66.0)	249 (66)	13 (65)	
Yes	135 (34.0)	128 (34)	7 (35)	
CHD, n (%)				0.405
No	310 (78.1)	296 (78.5)	14 (70)	
Yes	87 (21.9)	81 (21.5)	6 (30)	
cor pulmonale, n (%)				0.847
No	210 (52.9)	199 (52.8)	11 (55)	
Yes	187 (47.1)	178 (47.2)	9 (45)	
Heart.failure, n (%)				0.113
No	230 (57.9)	215 (57)	15 (75)	
Yes	167 (42.1)	162 (43)	5 (25)	
NEWS score category, n (%)				0.26
Low	38 ( 9.6)	38 (10.1)	0 (0)	
Medium	125 (31.5)	120 (31.8)	5 (25)	
High	234 (58.9)	219 (58.1)	15 (75)	
PaO2,(mmHg)	53.4 ± 16.4	53.3 ± 16.4	54.6 ± 17.3	0.73
PCO2,(mmHg)	59.7 ± 19.6	59.7 ± 19.5	60.2 ± 23.6	0.905
PH	7.4 (7.3, 7.4)	7.4 (7.3, 7.4)	7.3 (7.2,7.4)	0.074
WBC,(10*9)	8.6 ± 4.2	8.5 ± 3.8	12.0 ± 8.0	< 0.001
NEU%	77.6 ± 12.2	77.2 ± 12.3	85.2 ± 8.6	0.004
HB, (g/L)	135.1 ± 23.5	135.4 ± 23.6	128.2 ± 21.4	0.178
Platelet, (10*9)	209.8 ± 93.1	209.2 ± 90.6	219.7 ± 134.2	0.623
ALB, (g/L)	35.2 ± 4.7	35.3 ± 4.5	33.0 ± 6.3	0.033
BUN,(mmol/L)	6.5 (5.0, 9.0)	6.4 (4.9, 8.8)	10.1 (6.4, 14.4)	0.003
Creatinine, (umol/L)	63.5 (50.5, 81.3)	62.7 (50.2, 79.4)	100.4 (65.4, 123.7)	0.001
Potassium,(mmol/L)	4.1 ± 0.7	4.1 ± 0.6	4.2 ± 1.2	0.848
Sodium, (mmol/L)	139.3 ± 4.8	139.2 ± 4.7	140.8 ± 5.6	0.127

*Abbreviations: bpm* beat per minute, *SBP* Systolic Blood Pressure, *DBP* Diastolic Blood Pressure, *WBC* White Blood Countn *DM* Diabetes Mellitus, *Los* Length of stay, *ALB* Albumin, *CHD* Coronary heart disease, *BUN* Blood urea nitrogen, *HBP* High blood pressure, *WBC* White blood cell, *PaCO2* Partial pressure of carbon dioxide in arterial blood, *PaO2* Partial pressure of oxygen in arterial blood, *NEWS score* National Early Warning Score, *OR* Odds ratio, *CI* Confidence interval

**Fig. 2:**
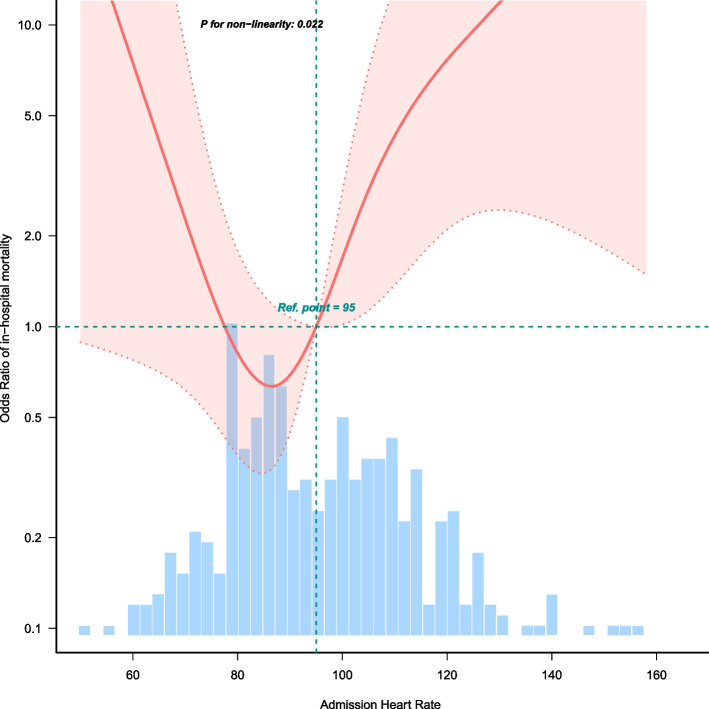
Curve fitting of admission heart rate and in-hospital mortality,*Adjustment factors included* age, sex, smoking status,NEWS score, Respiratory failure Type,DM,HBP,cor pulmonale,CHD, PO2,PCO2,NEU% HB,platelet,ALB,BUN,Creatinine,Potassium,Sodium

**Fig. 3:**
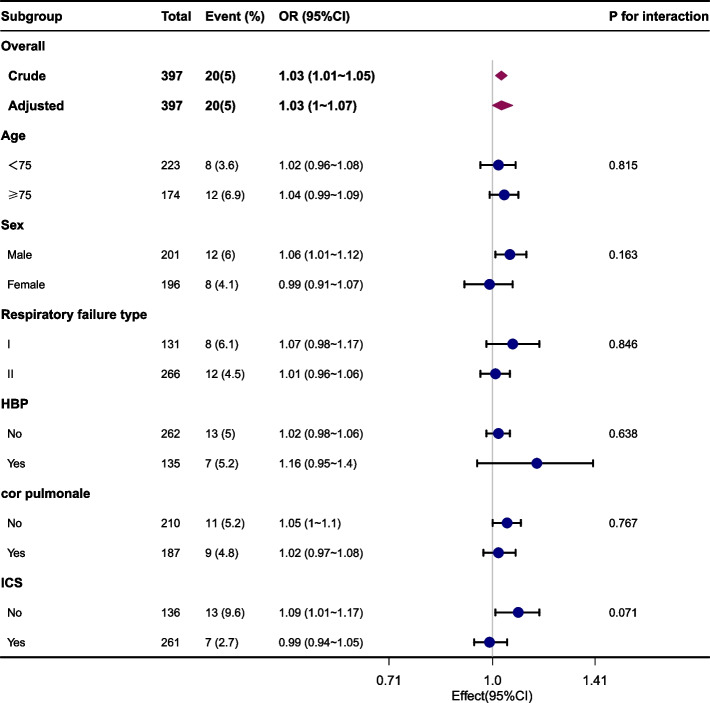
Stratified analysis of the association of admission heart rate on the risk of AECOPD in patients with Respiratory failure.*Adjustment factors included* age, sex, smoking status,NEWS score, Respiratory failure Type,DM,HBP,cor pulmonale,CHD, PO2,PCO2,NEU% HB,platelet,ALB,BUN,Creatinine,Potassium,Sodium. *Abbreviations :OR* Odds ratio, *CI* Confidence interval, *ICS* Inhaled corticosteroids

The association between AHR and in-hospital mortality was examined using univariate (Table 2) and multivariate (Table 3) logistic regression to assess the independent relationship between AHR and in-hospital mortality. As a continuous variable, AHR was associated with in-hospital mortality in univariate analysis (OR 1.03 [95% CI 1.01–1.05]; *p* = 0.012). AHR also remained an independent predictor of hospital mortality in model 1 which was non-adjusted (OR 1.03 [95% CI 1.01–1.05]; *p* = 0.012) and model 2 was adjusted for age, sex, smoking status, NEWS score (OR 1.03 [95% CI 1.01–1.05); *p* = 0.036), model 3, which was adjusted for the variables in model 2 plus RF type, DM, HBP, cor pulmonale, CHD (OR 1.03 [95% CI 1.01–1.05]; *p* = 0.035), model 4 which was adjusted for the variables in model 3 plus *P*O_2_, *P*CO_2_, NEU%, HB, platelets, BUN, ALB, creatinine, potassium, and sodium (OR 1.03 [95% CI 1.01–1.07]; p = 0.035). After adjusting for confounding factors, a 1 beat/min increase in AHR corresponded to a 3% increase in the risk for in-hospital mortality. Multivariate-adjusted restricted cubic spline analyses demonstrated a nonlinear relationship between AHR and in-hospital mortality (*p* = 0.022) (Fig. 2). Using threshold analysis, it was determined that the AHR threshold was 100 beats/min (Table 4). Above the threshold, each 1 beat/min increase in AHR had an effect size (OR 1.094 [95% CI 1.01–1.186]; *p* = 0.0281). Subgroup analysis was performed according to confounders, including age, sex, RF type, high blood pressure, cor pulmonale, and ICS, and the results were robust, and no significant interaction was found (all *p*-values for interaction > 0.05) (Fig. 3).

**Table 2:** Univariate analysis of risk factor associated with in-hospital mortality in Acute Exacerbation of Chronic Obstructive Pulmonary Disease with Respiratory Failure Patients

Variable	OR_95CI	*P*_value
Age	1.07 (1.01 ~ 1.13)	0.013
Sex		
Male	Ref	
Female	0.67 (0.27 ~ 1.68)	0.392
Los	0.79 (0.7 ~ 0.9)	< 0.001
Smoking status		
Never	Ref	
Former	1.53 (0.54 ~ 4.32)	0.425
Now	1.57 (0.48 ~ 5.09)	0.456
SBP	1 (0.98 ~ 1.02)	0.866
DBP	0.99 (0.96 ~ 1.02)	0.63
Heart rate	1.03 (1.01 ~ 1.05)	0.012
Respiratory rate	0.99 (0.83 ~ 1.19)	0.944
ICS		
No	Ref	
Yes	0.26 (0.1 ~ 0.67)	0.005
Ventilator		
No	Ref	
Yes	0.23 (0.08 ~ 0.71)	0.01
Respiratory failure type		
I	Ref	
II	0.73 (0.29 ~ 1.82)	0.496
DM		
No	Ref	
Yes	0.91 (0.2 ~ 4.07)	0.902
HBP		
No	Ref	
Yes	1.05(0.41 ~ 2.69)	0.923
CHD		
No	Ref	
Yes	1.57 (0.58 ~ 4.2)	0.373
cor pulmonale		
No	Ref	
Yes	0.91 (0.37 ~ 2.26)	0.847
Heart failure		
No	Ref	
Yes	0.44 (0.16 ~ 1.24)	0.122
NEWS score	1.44 (1.18 ~ 1.75)	< 0.001
PO2	1 (0.98 ~ 1.03)	0.729
PCO2	1 (0.98 ~ 1.02)	0.904
PH	0.01 (0 ~ 0.31)	0.01
WBC	1.14 (1.05 ~ 1.23)	0.001
NEU%	1.08 (1.02 ~ 1.14)	0.005
HB	0.99 (0.97 ~ 1.01)	0.177
Platelet	1 (1 ~ 1.01)	0.622
ALB	0.9 (0.82 ~ 0.99)	0.034
BUN	1.1 (1.04 ~ 1.17)	0.001
Creatinine	1.01 (1.01 ~ 1.02)	< 0.001
Potassium	1.07 (0.55 ~ 2.06)	0.847
Sodium	1.09 (0.98 ~ 1.21)	0.121

*Abbreviations: bpm* beat per minute, *SBP* Systolic Blood Pressure, *DBP* Diastolic Blood Pressure, *WBC* White Blood Count, *DM* Diabetes Mellitus;Los:Length of stay, *ALB* Albumin, *CHD* Coronary heart disease, *BUN* Blood urea nitrogen, *HBP* High blood pressure, *WBC* White blood cell, *PaCO2* Partial pressure of carbon dioxide in arterial blood, *PaO2* Partial pressure of oxygen in arterial blood, *OR* Odds ratio, *CI*, Confidence interval, *ICS* Inhaled corticosteroids, *NEWS score* National Early Warning Score

**Table 3:** Association between admission Heart rate and in-hospital mortality in multiple regression

Variable	Model 1		Model 2		Model 3		Model 4	
	**OR_95CI**	***P*_value**	**OR_95CI**	***P*_value**	**OR_95CI**	***P*_value**	**OR_95CI**	***P*_value**
Heart rate(bpm)	1.03 (1.01 ~ 1.05)	0.012	1.03 (1.0 ~ 1.05)	0.036	1.03 (1.0 ~ 1.05)	0.035	1.03 (1.0 ~ 1.07)	0.035

Mode 1: Non-adjusted

Model 2: Adjust for age, sex, smoking status,NEWS score,

Model 3: Adjust for the variables in Model 2 plus Respiratory failure Type, DM,HBP,cor pulmonale,CHD,

Model 4: Adjust for the variables in Model 3 plus PO2,PCO2,NEU%, HB,platelet,ALB,BUN,Creatinine,Potassium, Sodium,

*Abbreviations:*OR Odds ratio, *CI* Confidence interval

**Table 4:** The nonlinear relationship between admission heart rate and in-hospital mortality

Threshold of heart rate	OR	95%CI	*P-vale*
< 100	**0.474**	**0.016 ~ 13.683**	**0.6635**
≥ 100	**1.094**	**1.01 ~ 1.186**	**0.0281**
Likelihood Ratio test			**0.019**

*Adjustment factors included* age, sex, smoking status,NEWS score,Respiratory failure Type,DM,HBP,cor pulmonale,CHD, PO2,PCO2,NEU% HB,platelet,ALB,BUN,Creatinine,Potassium,Sodium

*Abbreviations: OR* Odds ratio, *CI* Confidence interval

## Discussion

The present study analyzed the relationship between AHR and in-hospital mortality in patients with AECOPD and RF admitted to a hospital ward. AHR was found to be independently associated with in-hospital mortality. Furthermore, a non-linear relationship was observed in restricted cubic spline analysis of the association between AHR and in-hospital mortality in our study population, indicating an inflection point at approximately 100 beats/min. For AHR ≥ 100 beats/min, we found that each 1 beat/min increase in AHR corresponded with an increase in risk for in-hospital death by 9.4%. In addition, there were no significant differences of survival among the patients with heart rate < 100 beats/min.

Many studies have shown that increased resting heart rate is a major risk factor for cardiovascular disease [20, 21] and is associated with cardiovascular and all-cause mortality [22–24]. It has been reported that there are more cardiovascular events in patients with non-ST-segment elevation acute coronary syndrome at a high heart rate [25]. A study showed that a heart rate ≥ 120 beats/min risk factors associated with in-hospital death from coronavirus disease 2019 [26]. Heart rate is an independent predictor of in-hospital death in patients with intermediate- to high-risk acute pulmonary embolism [27]. Therefore, heart rate has been confirmed as an important determinant of adverse events in patients with cardiovascular disease [28]. Chen et al. indicated that an AHR > 90 beats/min was an independent predictor of short-and long-term mortality in patients with acute aortic dissection [29]. Okuno et al. found that AHR was a determinant of the effectiveness of beta-blockers in patients with acute myocardial infarction [30]; however, the association between AHR and in-hospital mortality of patients with combined AECOPD and RF was unclear.

AECOPD considerably affects disease progression, worsens pulmonary function, increases the risk for further exacerbation and death, and impairs quality of life [31, 32]. Studies have reported that the in-hospital mortality rate for AECOPD is 4.8%–10.4% [33]. Morasert et al. indicated that respiratory failure on admission was a prognostic indicator of in-hospital mortality in patients with AECOPD [34]. Simultaneously, patients may have a negative impact on the short term survival, who had an obvious respiratory failure [35]. Many studies have probed the risk factors associated with mortality in patients with AECOPD, to date, it is known that RR, BUN, blood gas analysis (Hb, *P*CO_2_), ALB are inflammation-related indicators, are important prognostic factors for mortality in these patients [36], and our results are consistent with these results. Combined with clinical practice, we hypothesize that AHR may be associated with in-hospital mortality in patients with AECOPD and RF, although no relevant study has focused on the association between them. The present study investigated the association between AHR and in-hospital mortality in AECOPD patients with RF who were admitted to hospital and found a significant association. An AHR of 100 beats/min was identified through a two-piecewise linear regression model; as such, results of this study may be helpful for respiratory physicians to select appropriate medical and interventional measures according to AHR.

The exact mechanisms underlying the relationship between AHR and clinical outcomes in AECOPD patients with RF are not well known, although potential explanations include the following. Sympathy-vagal imbalance, with autonomic dysfunction in COPD, may be the main factor for elevated heart rate [37]. Rapid heart rate may be a risk marker of autonomic imbalance, which may directly promote myocardial ischemia or heighten the potential for arrhythmias or sudden death [38]. Particulate matter in polluted air and cigarette smoke have been reported to be associated with autonomic dysfunction [39, 40]. Furthermore, nicotine in cigarette smoke can increase sympathetic activity [41]. Another reason for autonomic dysfunction could be chronic hypoxemia. Simultaneously, autonomic dysfunction has been associated with arrhythmia and sudden cardiac death [42]. Compensation for elevated heart rate may be caused by the mechanical effects of obstruction and hyperinflation on cardiac filling, which may be an important factor [43, 44].

Our study had several strengths. The data demonstrated that AHR may be used as an easily obtained risk marker to predict the prognosis of patients with AECOPD and RF. Furthermore, AHR is a convenient indicator for identifying high-risk AECOPD in patients with RF and can help physicians assess the state of illness.

However, the present study also had some limitations, the first of which was its retrospective design and that we could not obtain all baseline characteristics,such as the first heart rate in the emergency department for patients admitted in an emergency, which may have led to biased results. Second, we could not assess whether the excluded patients had an impact on the results of this study. We did not collect data regarding other factors associated with AECOPD/RF mortality, including lung function, long-term oxygen treatment, body mass index,and globulin. We also did not collect data regarding the use of β-blocker(s), vasopressors,which may affect heart rate. Third, our investigation was a single-center study, and the sample size was small; therefore, so the possibility of selection bias and lack of a validation cohort cannot be ruled out. Finally, heart rates in these patients were evaluated only on admission, and no dynamic analyses of the association between heart rates over time and patients with AECOPD/RF were performed. As such, future studies should be designed to address these limitations in an effort to validate and expand on our results.

## Conclusion

AHR was associated with increased all-cause in-hospital mortality in patients with AECOPD and RF. Therefore, as a simple and accessible parameter, an elevated AHR should be a risk signal to alert respiratory physicians to perform intervention(s) early.

## Data Availability

The datasets generated and/or analyzed in the current study are available from the corresponding author upon reasonable request.

## Abbreviations

AECOPD: Acute exacerbation of chronic obstructive pulmonary disease
AHR: Admission heart rate
ALB: Albumin
BUN: Blood urea nitrogen
Hb: Hemoglobin
CHD: Coronary heart disease
DM: Diabetes mellitus
GOLD: Global Initiative for Chronic Obstructive Lung Disease
ICS: Inhaled corticosteroid(s)
NEU: Neutrophils
*Pa*CO_2_: Partial pressure of carbon dioxide
*Pa*O_2_: Partial pressure of oxygen
RF: Respiratory failure
RR: Respiratory rate
